# The challenge of screen mediated shared reading for children’s learning and engagement

**DOI:** 10.3389/fpsyg.2023.1212173

**Published:** 2023-07-27

**Authors:** Kirsten Read, Hayley Harrison, Brianna Mireku, Katrina Ying

**Affiliations:** Department of Psychology, Santa Clara University, Santa Clara, CA, United States

**Keywords:** shared reading, preschoolers, video-chat, word learning, engagement

## Abstract

**Introduction:**

Screen mediated shared reading (SMSR), which involves an adult reading a child a book through video-chat, is a recent development in shared reading. In this study, we investigated whether, as in in-person shared reading, children could learn new words from SMSR, and whether having a physical copy of the book to follow along with impacted children’s novel word retention and engagement in this setting.

**Method:**

Three- to 5-year-old participants (*n* = 34) were read an 8-page rhyming, “Meet the Friendly Monsters” story by a researcher over Zoom, via screen sharing an e-version of the story used in previous studies. Participants were randomly assigned to one of two conditions: SMSR with or without a paper copy of the book to follow along with. The session was recorded to measure children’s engagement behaviors during the reading. Novel word learning from the story was measured with both a multiple choice identification test and a monster naming test, both administered immediately after the initial reading, then re-administered 10–14 days later. Engagement during the SMSR sessions was measured every 30-s on a scale of 1 (low engagement) to 5 (high engagement), and then averaged for each child.

**Results:**

Results show that children overall performed slightly above chance on novel monster name retention from the SMSR. However, retention was not as strong as seen in other in-person shared reading studies using the same story. Additionally, while children remembered monster names with equal efficacy regardless of condition and level of engagement, there were still qualitative differences in the reading sessions depending on whether children had their own paper copy of the book to follow along with – in general, the ‘with book’ condition appeared to *increase* the challenges posed to children’s attention during SMSR, potentially making the word learning task more difficult.

**Discussion:**

The findings of this study have implications for how to approach shared reading with young children in new contexts with the use of emerging technology. We raise future research questions for a better understanding of best practices for screen mediated shared reading.

## Introduction

### Learning from shared reading

Shared book reading, which involves an adult caregiver reading, and a child listening and actively participating in children’s book read-alouds, has long been considered beneficial for preschool-aged children’s linguistic and socio-emotional learning (Yuill and Martin, 2016; Neuman et al., 2020; Şimşek and Ergodan, 2020). Learning from shared reading can result, at least in part, from increasing the interactivity and engagement of the child (e.g., Lingwood et al., 2021). However, this raises the question of how best to both encourage and measure engagement in shared reading, and within different types of shared reading contexts. Interactive shared reading, in which the child and reader are both visually engaged with the book, sharing attention, and actively commenting on it, supports learning for preschool children (e.g., Farrant and Zubrick, 2013; Şimşek and Ergodan, 2020). Recent research on the use of screen-based modalities (i.e., e-books) for shared reading at home and in the classroom has pushed the question of how to support children’s engagement in reading into this emerging new sphere. Previous e-book reading studies have explored the balance between including features that promote children’s engagement to encourage learning versus avoiding novel features that could potentially distract children from learning (Takacs et al., 2015; Rvachew et al., 2017; Revelle et al., 2019).

This work highlights both the positive relationship between engagement and learning as well as the nuances of measuring engagement and learning in these contexts (Lauricella et al., 2014; Rees et al., 2017). In a study comparing print book to e-book reading with 4-year-olds, Lauricella et al. (2014) successfully measured dyadic (parent *and* child) engagement with 1–3 point scales of parent involvement, mutuality of communication, parent initiated story engagement and turn taking, and found both that parent engagement was higher during e-book reading, but also that parent–child engagement positively correlated with children’s story comprehension. In a more recent study, Lingwood et al. (2021) expanded on the existing research on children’s engagement during shared reading by using the dynamic measurement of engagement across a traditional parent and child print book shared reading experience to quantify how children’s level of engagement naturally fluctuates throughout a story, finding correlations between the behaviors of the adult readers and moments of high engagement for children. The definition of engagement varies across the literature, but generally speaking, high engagement on the part of the child involves concentration, attention to detail, active imagination and thought, and this may be demonstrated by comments, questions, or responses to the book by the child (Lingwood et al., 2021). In a recent study by Şimşek and Ergodan (2020) dialogic reading, i.e., when the reader goes beyond the text of a book to encourage a child’s participation through questions and prompts, was found to increase interactions by the child; and thus they posit that dialogic reading, above and beyond the modality of traditional print book reading vs. digital reading, increased children’s engagement, which resulted in significantly improved language development.

While there are many potential benefits of shared reading for young children and learning outcomes from a read-aloud that can be measured, such as emergent literacy skills (Parish-Morris et al., 2013; Rvachew et al., 2017), or story comprehension (Lauricella et al., 2014), in this study we will focus on learning new words from a read aloud. For preschool-aged children, research measuring language development often focuses on vocabulary development and the ability for the child to retain novel words from the stories that they hear (Read, 2014; Read and Quirke, 2018; Gaudreau et al., 2020). Research has shown that children can learn novel words from shared reading, and even “fast map” new words from a single read aloud (e.g., Read, 2014; Read and Quirke, 2018; Neuman et al., 2020). However, given the complexities in measuring children’s engagement, and the rapid emergence of new modalities for shared reading, research has yet to fully examine the connection between dynamic engagement and fast-mapping new words from shared reading in new contexts. Thus, to expand on prior research showing a positive relationship between engagement and learning, we aim to test the correlation between children’s engagement during shared reading and their subsequent fast mapping of new words from a single read aloud in a fast-emerging screen mediated modality.

### Shifts in shared reading mediums

The growing prevalence of e-reading in the past decade has allowed for more research on its effectiveness not only in isolation, but also relative to more traditional print-book reading in terms of children’s retention, engagement, and interaction in shared book reading settings (Revelle et al., 2019; Gaudreau et al., 2020). To understand children’s engagement during shared reading is to understand the context in which the shared reading experience occurs. Recently, this context has increasingly included varying electronic or e-reading options. Prior to 2020, the transition to e-reading largely consisted of electronic books accessed through a tablet or computer. This e-reading involves the reader and child still in the same location, but with text being shifted from a paper to a digital medium. While some prior research has included video-chat settings, most e-reading studies have not incorporated screen mediated shared reading, i.e., synchronous reading with a live reader and book not physically present but viewed via a screen (e.g., using FaceTime or Zoom), which has become more common especially in educational settings since the pandemic (Read et al., 2021; Strouse et al., 2021).

### The acceleration of SMSR as a result of the pandemic

Due to school closures and social distancing in 2020, preschool children as young as 3 and 4 were required to grow accustomed to the implementations of technologies such as video-chat into their daily learning, and even specifically in their at-home reading routines (Read et al., 2021). This included the replacement of a caregiver or teacher reading a physical book to preschoolers in a traditional educational setting with the possible solution of playing back a recorded reading of a book, use of an e-book app, or even live video-chat based shared reading (e.g., virtual library storytime, or reading over FaceTime with distant family member). This has come to be known as screen mediated shared reading (SMSR), and there has since been an increase in these methods (Gaudreau et al., 2020; Read et al., 2021). In a recent study, 31% of grandparents of children under 5 reported reading aloud with their grandchildren over video-chat interactions when they could not be together in physical proximity (Strouse et al., 2021). This increased use of SMSR has undoubtedly led to impacts on the shared reading experiences between distant caregivers and children (Furenes et al., 2021).

In one of the first studies to consider the specific context of screen mediated reading, Gaudreau et al. (2020) compared comprehension and word learning during shared reading with a group of 4-year-old preschoolers in three different settings: with a live experimenter, with an experimenter on video-chat, or with an experimenter in a prerecorded video. Children’s story comprehension did not significantly differ between the live and video-chat settings (Gaudreau et al., 2020). When comparing digital reading, in which the book was digitally cast through a projector without the use of a physical book, to traditional reading in classrooms over many weeks, Şimşek and Ergodan (2020) also found there was no major influence of digital reading on 48- to 66-month-olds’ language development according to scores on the Test of Early Language Development (TELD-3). Thus, preschool aged children are able to understand a book comparably when shared reading occurs in person traditionally and via a screen.

Gaudreau et al. (2020) and Şimşek and Ergodan (2020) found that general comprehension remained largely unaffected by shifts in mediums toward electronic reading. However, the more stringent test of learning – being able to fast map a novel word presented for the first time in a read aloud – is a harder task for young children, and may be more challenging in a screen mediated environment. Testing this more challenging word learning task may help us better understand the extent to which different e-reading modalities impact language development in young children, and the role that different levels of engagement might play in learning from shared reading. As highlighted by Morini and Blair (2021), there are many factors to consider when collecting data remotely, including the duration of the session and the amount of distractions around the child.

The increased utilization of live video-chat reading as a result of the pandemic has created an elevated focus on research involving screen mediated shared reading in recent years. Emerging research from Shields et al. (2021) has shown that virtual interactions via video-chat are effective for children’s learning. There are some concerns regarding research conducted via video-chat as such settings tend to be more varied and distracting for children and produce challenges to observations and data collection (Chuey et al., 2021; Morini and Blair, 2021). However, Chuey et al. (2021) found that participant sessions that are moderated by a live experimenter maintain the interactive experience of in-person studies. For research and classroom settings alike, synchronous, live SMSR can be the most effective e-reading method (Chuey et al., 2021). There is, however, a gap of knowledge around the limitations of SMSR, resulting in the continued need to understand the differences between SMSR and traditional in-person reading. Even as in-person learning has re-commenced, SMSR is not likely to go away. Therefore, the present research focuses on this new medium, which has emerged as a prevalent e-reading method in recent years.

### Benefits of physical books

The transition to more frequent screen mediated reading typically eliminates physical books from a shared reading experience in favor of e-books. The removal of physical books is a unique condition of screen mediated reading, that adds a new aspect to the shared reading experience for children. However, research has shown benefits of the presence of a physical book for children in a shared reading setting (Yuill and Martin, 2016). When children have more autonomy and control in an activity like shared reading they tend to be more engaged with the content provided (Roskos et al., 2012). When comparing child-led to mother-led shared reading, Yuill and Martin (2016) rated children, aged 7–9 years, higher on an engagement scale from one to five based on visual attention, gestures, expression, and verbalization that were coded per minute of each reading session. Child-led shared reading involves the child sounding out and verbalizing the story, and oftentimes involves the child controlling page turning and holding of the physical book or tablet (Yuill and Martin, 2016). In their study, regardless of child-led or mother-led reading, the reader favored a physical book over an e-book. While this analysis was largely impacted by an increase of maternal comments in the paper book condition and not a direct measure of children’s engagement, Yuill and Martin suggest that their findings were due to a greater opportunity for, and thus greater amounts of, engagement during shared reading with a paper book compared to an e-book. Children’s opportunities to directly assert control over books has been found to increase engagement, such as when children are able to physically flip the pages of a book (Raffle et al., 2010) or even control the mouse in an e-reading environment (Calvert et al., 2005). Thus, physical interaction with books may be an important aspect of holding children’s attention during shared reading.

Critically, shared reading with paper books may offer less distraction than synchronous reading over video-chat. According to Shields et al. (2021), environmental distractions are more frequent for research conducted remotely. For this reason, shared reading over video-chat may be more attentionally demanding. If physical books may better enable engagement compared to e-books, then a child being able to hold and control their own physical copy of a book while participating in a screen mediated reading session may help them maintain their attention.

### The present research

Given this prior research on the ability of children to learn new words from shared reading experiences, and the rapid advent of a new medium for engaging with and reading with young children remotely – screen mediated shared reading, the aim of the present research is to address the following questions:

1. Can children fast map and remember new words from screen mediated shared reading (SMSR)?
2. Does the addition of a physical book influence the extent to which children can fast map new words from SMSR?
3. Does the addition of a physical book influence children’s engagement during SMSR?
4. Does children’s engagement during SMSR predict their ability to fast map new words from a story?

## Method

The experimental protocol described below was approved by the Santa Clara University Institutional Review Board for Social Behavioral Educational Research (protocol ID: 21–04-1,618). Consent was obtained from the guardian of each participant before any information was collected. The hypotheses and analysis strategy were pre-registered with AsPredicted (#66228).

### Participants and design

Participants (*n* = 34) between the ages of 3–5 years (*M_age_* = 52 months, range: 38–68 months) completed the study. They were identified as 13 male and 21 female, and all were English dominant. One participant completed only the first session of the study, but data from the first session is included in the results. We did not collect information regarding race, ethnicity, SES, or other demographic factors.

Participants were recruited via email from parents’ groups, our lab’s database of families who have previously indicated interest in research participation, and broader social media posts seeking preschool-aged participants for a remote shared reading research study. Criteria for participation in the study was English as the primary language (used at least 50 percent of the time at home), and typical learning ability (i.e., no learning disabilities) to ensure comprehension of the story and the questions asked in the procedure. All participants had a level of familiarity with shared reading and with some form of video-chat, but not necessarily a combination of the two as utilized by our study. In a between-subjects design, participants were randomly assigned to one of two conditions – a screen mediated story reading (SMSR) with access to a physical book vs. a SMSR without an additional physical book.

### Materials

This study employed the same “Meet the Monsters” storybook that has previously been used in experimental research on vocabulary learning from shared reading, and that children in this age range have demonstrated that they understand and enjoy (e.g., Read, 2014; Read et al., 2019, 2021). The book was created in-house, and is not commercially available, so that it would be novel to all participants, in order to control any familiarity effects. The story introduces children to eight new monster names. Each page of the story depicts one monster and a rhyming stanza introducing the novel monster’s name.

**Figure 1:**
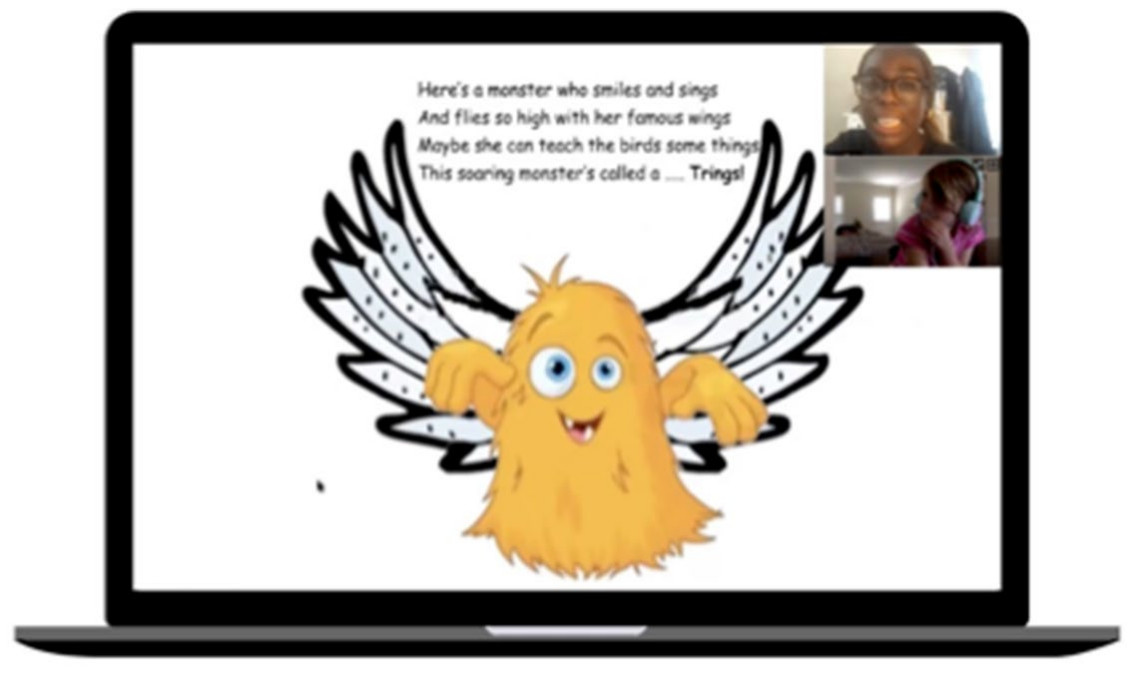
Example of screen set-up during SMSR session.

The layout of both the on-screen version and the paper copy of the book were the same – a centered illustration of each monster on a white background with the text of the rhyme placed individually on each page. The physical copy was printed on glossy, 8 × 10 inch photo paper in landscape orientation to look and feel like a typical softcover children’s book that closely matched the screen version. See Figure 1 for an example page and screen set-up.

We utilized the video-chat platform Zoom to conduct each session of the study. Zoom allows for live presentations by the research assistant over video-chat, while simultaneously sharing their screen to show the “Meet the Monsters” book. Since the pages of the story were already created as individual slides using PowerPoint software, the screen share function was utilized to share the storybook and the related retention measures for all conditions, while both the video of the reader (researcher) and the child’s video were also visible.

### Procedure

After obtaining informed consent and basic background information from the caregiver and assent from the participant, the study commenced with the first of two video-chat sessions. The first session typically lasted about 10 min. Participants had Zoom downloaded prior to participating in the study. Caregivers were instructed to limit any interaction and commentary during the reading. The researcher shared their screen via the video-chat platform to show the “Meet the Monsters” book to the participant. The researcher introduced herself, welcomed the child, and obtained verbal assent from the child that they were ready to hear a story and then answer some questions about it. The researcher then proceeded to read the book to the child, forwarding the screen to the respective page as they read aloud the rhymes describing and naming each of the eight novel monsters in turn. The same female researcher read the same book in the same age-appropriate reading style throughout the story for all participants in both conditions.

In the ‘without book’ condition, the reader shared the story on a screen and read it to the child over video-chat. In the ‘with book’ condition, the reader shared the story on a screen, and the child had their own physical copy of the book. Participants in the ‘with book’ condition were asked if they had their book present for the reading, but were not given further instructions regarding how to use or interact with the physical book. At the completion of session 1, participants in the ‘with book’ were instructed to have no interaction with the storybook until the completion of session 2 which would take place 10–14 days later.

Following the reading of “Meet the Monsters,” the child completed two short monster name retention tests – first, a multiple choice test that measured novel word identification, in which participants were tested on their ability to identify a particular monster image according to the novel name provided by the researcher; and second, a monster name recall test that measured novel monster name production, which tested participants’ ability to verbalize the monster name of a provided monster image. Approximately 10–14 days later, a second video-chat session was held in which the researcher conducted the same retention tests, without reading the story. Since session 2 did not include as much introduction from the researcher or a reading of the story, it typically only lasted a few minutes with each participant. At the end of the second session parents and children were thanked for their participation and sent an electronic gift card to an online children’s book retailer.

### Measures of retention and learning

Novel word retention and learning were measured through accuracy on monster identification and production tests. These tests were adapted from previous studies to a powerpoint presentation that was shared during the Zoom session (Read et al., 2019). Similar to previous studies, to measure identification, children were shown eight slides with pictures of the monsters and asked to point to or describe their chosen target (e.g., “Which one is the Smooze?”). In this study, the identification test used showed 4-alternative choices of monster pictures per slide. Each monster was targeted once during the eight trials after the first storybook reading and again after the second reading. Children’s identification scores were a simple calculation of the number of correctly identified monsters out of the eight multiple choice questions, with scores ranging from 0 to 8.

To measure production, the same procedure from previous studies was used in both the first and second session. Participants were shown an additional eight slides, each with a picture of a single monster, and asked “What’s this monster called?” Children received one point for each monster correctly named, and a half-point if they gave a monster name that was a close approximation (e.g., “schmooze” instead of “smooze”), again resulting in possible scores ranging from 0 to 8.

In all cases higher scores indicated participants’ greater success in remembering the monster names heard within the story and a greater potential for long-term novel word retention and learning.

### Measures of engagement

Participants’ behavior throughout the shared reading was coded using an adapted version of the Leuven Scale of Active Engagement to better comply with the screen mediated setting of this study (Laevers, 2008; Lingwood et al., 2021). The Leuven Scale of Active Engagement measures how engaged a child is during an activity on a scale from 1 (extremely low engagement) to 5 (extremely high engagement). A child who displays low engagement (1 or 2) might show vacant staring, looking down, no facial expression, or looking and listening, but without much reaction. A child who displays high engagement (4 or 5) reacts to the story in at least one way, e.g., with a verbal comment, some facial expression, or other physical gesture.

The original Leuven Scale of Active Engagement was created for in-person tasks to be completed by children in the presence of a researcher or parent. This study used an updated dynamic measurement version of the Leuven Scale of Active Engagement (Lingwood et al., 2021), which involved rating the child on the scale in 30 s increments during the reading. Because of differences between in-person activities and SMSR, including the configural limits to joint visual attention (i.e., reader and child cannot sit side-by-side and look at the same thing), changes to the scale to accurately measure engagement during reading in a video-chat setting were updated to focus more on the child’s active response to the book, rather than simply their visual attention.

Researchers measured engagement from the recorded reading session, attending to the following behaviors: verbal comments and questions from the children, looking at the screen or away, nonverbal gestures and facial expressions that were responsive to the story being read aloud. Two coders independently watched each recording and coded the participants’ engagement behavior, then met to discuss any discrepancies in coding in order to converge on a single score.

To normalize scores across the varying lengths of reading time, each participant was assigned a single engagement score derived from the average of scores across their session at the completion of the coding session. The average length of storybook reading was 164.7 s (range: 150–330), or just under 3 min across the 34 participants. When divided into 30-s increments for engagement coding, this meant children’s mean engagement score was derived from an average of 5.5 increments (range: 5–11). Participants were given an engagement score for the last increment range, even if the increment did not last a full 30 s (i.e., if a participant read for 2 min and 40 s, the child was assigned engagement scores for 6 increments).

## Results

### Participant factors

In order to understand which individual participant factors may be relevant to children’s responses in this study, we conducted exploratory analyses on the impact of participants’ gender and age (in months) on each of the three main outcome variables – total identification scores across both sessions, total production scores across both sessions, and engagement averages while listening to the stories. We found no effect of gender on any of these outcome measures (*p*-values all >0.44, *d*’s all <0.30), and subsequently did not include gender as a predictor variable in any of the analyses described below. Age, however, was positively correlated with children’s total identification scores (*r* = 0.38, *p* = 0.028) and their total production scores (*r* = 0.45, *p* = 0.009). As may be expected, older children tended to be more successful in their retention of the new monster names, although age was not correlated with participants’ average engagement scores (*r* = −0.18, *p* = 0.300). Age was, therefore, included as a covariate in the relevant analyses of children’s retention that follow.

### RQ1: Can children fast map and remember new words from SMSR?

In order to address the first research question of this study, both the mean identification scores and mean production scores of all 34 participants were compared to chance levels of responding after both the first shared reading session and after the second. For identification scores, chance performance was set at 2 correct out of 8 (or a score of 2.0) on a 4-alternative multiple choice test. One sample *t*-tests showed that after hearing the monster story read once, participants’ identification scores (*M* = 2.441, *SD* = 1.52) were significantly greater than chance, *p* = 0.050, but this effect was small, *d* = 0.29, and approximately 2 weeks later, participants’ identification scores decreased (*M* = 2.030, *SD* = 1.33) to a level no longer different from chance, *p* = 0.499, *d* = 0.02.

Children’s mean production scores were tested against 0 in order to know whether there was any evidence that children recalled the novel monster names across conditions. One sample t-tests showed that after hearing the monster story read once, participants’ production scores (*M* = 0.25, *SD* = 0.51) were significantly greater than 0, *p* = 0.004, *d* = 0.49, and approximately 2 weeks later, participants’ production scores increased (*M* = 0.53, *SD* = 0.87) and were also significantly different from 0, *p* < 0.001, *d* = 0.61.

In sum, in the simpler monster name identification task, participants recognized the novel monsters better than chance immediately after hearing the story shared over video-chat, but not a couple weeks later. At both time points, in the production task participants were able to recall monsters correctly more often than *never*, but that may be a low bar for contending that they were learning the novel words. Thus, while objectively we can say that children remembered something of the monster names after hearing them read aloud via screen mediated shared reading, their fast mapping and retention of these new labels was not strong or definitive.

In addition, to better contextualize children’s retention in this study, a comparison of the effect sizes found in the current study can be made with those from previous studies of retention of monster names using this same storybook shared during in-person reading. In previous studies from our own lab that used the same monster story stimuli and similar tests of monster name retention via a multiple choice identification task, we found effect sizes when comparing children’s performance on monster name identification to chance of *d* = 0.64 (Experiment 1), *d =* 1.21 (Experiment 2, Read, 2014), and *d* = 1.18 (Read et al., 2019); and even recently, *d* = 2.12 (Read et al., 2022) in a study where participants read the monster book at home with their caregivers repeatedly over a few days. While other conditions in previous studies vary in ways that make direct statistical comparison impossible (e.g., different amount of exposure to the stories, different conditions, different testing protocols), it is still worth noting that with the same monster story and same types of retention tests, in the current study children’s retention relative to chance was at the low end of the range of effect sizes we have seen in other studies that presented these stories in in-person contexts.

### RQ2: Does the addition of a physical book influence the extent to which children can fast map new words from SMSR?

Despite children’s underwhelming ability across conditions to remember the newly introduced monster names in this screen mediated shared reading protocol, it was still of interest whether we might see differences between the two reading conditions. Thus, in order to test whether the condition that children participated in (with or without their own paper copy of the book for following along) impacted their retention, first an omnibus 2 × 2 analysis of variance (ANOVA) was conducted on identification and on production scores with condition (with or without paper book) as a between-subjects factor, and session (first or second test session) as a within-subjects factor, and age in months as a covariate.

**Figure 2:**
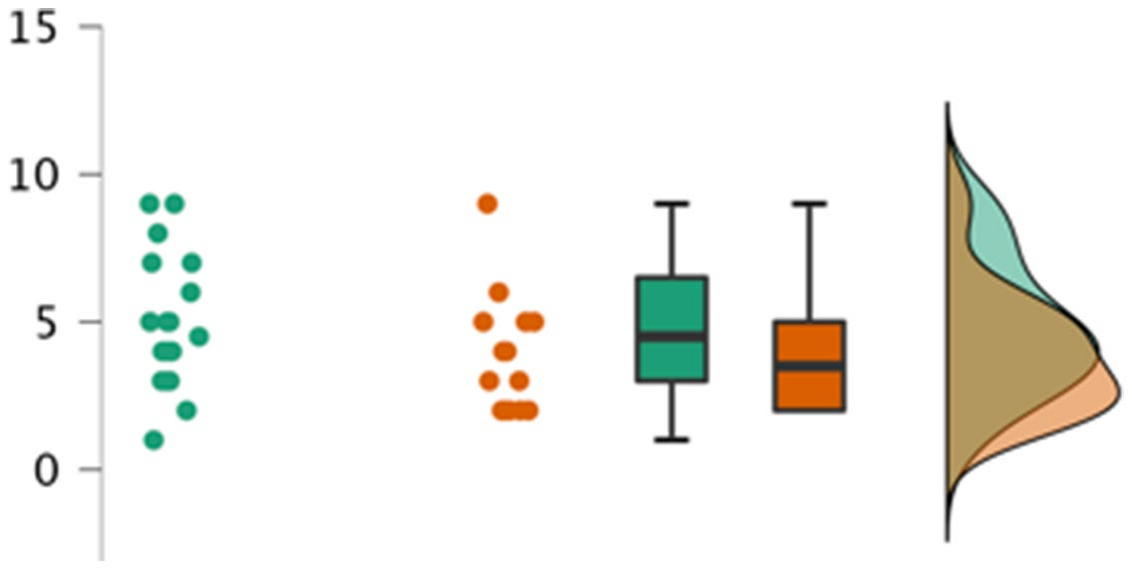
Participants’ total identification scores (across both sessions) by condition. In three alternative ways the distribution of identification scores for participants are represented with dots, box plot, and curves. Green indicates the scores of participants in the ‘without book’ condition; Orange indicates scores for participants in the ‘with book’ condition.

Participants’ identification scores in each condition at each testing session are shown in Table 1. The ANOVA on these retention scores revealed that there was a significant main effect of age, *F*(1, 30) = 7.362, *p* = 0.011, η^2^ = 0.11; a main effect of condition only approaching significance, *F*(1, 30) = 3.697, *p* = 0.064, η^2^ = 0.05, suggesting marginally higher identification scores in the without book condition; and no significant main effect of session, *F*(1, 30) = 0.013, *p* = 0.909, η^2^ < 0.001. In addition, there were no significant interactions between condition and session, *F*(1, 30) = 0.105, *p* = 0.748, η^2^ = 0.001, between age and condition, *F*(1, 30) = 0.014, *p* = 0.912, η^2^ < 0.001 or between age and session, *F*(1, 30) = 0.082, *p* = 0.776, η^2^ = 0.001. See Figure 2 for a detailed depiction of the distribution of total identification scores (summed across test sessions) by condition.

**Table 1:** Identification score means (standard deviations in parentheses) by condition and test session.

Condition	Session 1	Session 2	Total across sessions
Without book	2.70 (1.49)	2.21 (1.40)	4.87 (2.28)
With book	2.07 (1.54)	1.79 (1.25)	3.86 (2.03)

**Figure 3:**
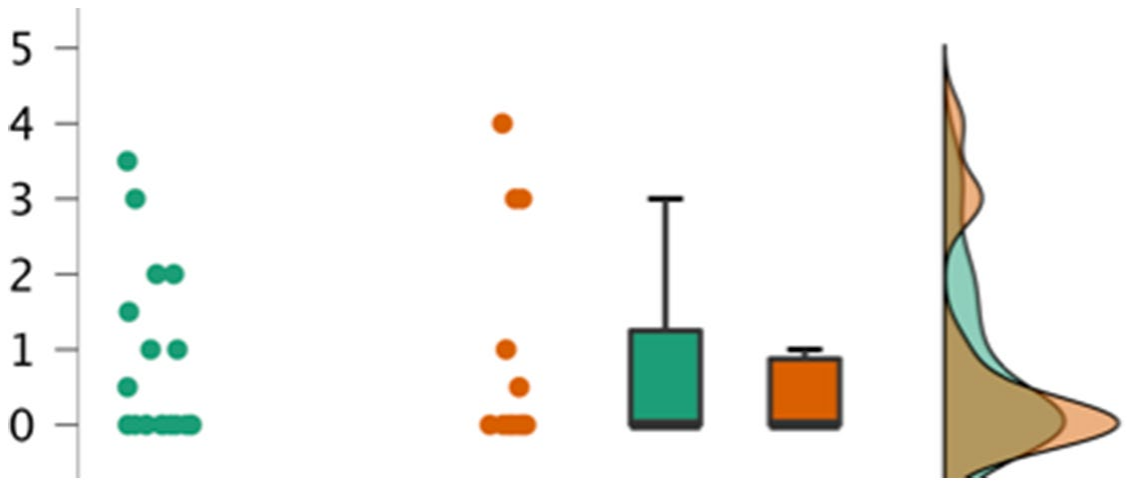
Participants’ total production scores (across both sessions) by condition. In three alternative ways the distribution of production scores for participants are represented with dots, box plot, and curves. Green indicates the scores of participants in the ‘without book’ condition. Orange indicates scores for participants in the ‘with book’ condition.

Participants’ production scores in each condition at each testing session are shown in Table 2. The ANOVA on these retention scores revealed that there was again a significant main effect of age, *F*(1, 30) = 7.513, *p* = 0.010, η^2^ = 0.15, but no significant main effects of condition, *F*(1, 30) = 0.104, *p* = 0.749, η^2^ = 0.002; or session, *F*(1, 30) = 0.153, *p* = 0.698, η^2^ = 0.001. There were also no significant interactions between condition and session, *F*(1, 30) = 0.040, *p* = 0.843, η^2^ < 0.001; condition and age, *F*(1, 30) = 0.139, *p* = 0.713, η^2^ = 0.001; or session and age, *F*(1, 30) = 0.519, *p* = 0.477, η^2^ < 0.004. See Figure 3 for a detailed depiction of the distribution of total production scores (summed across test sessions) by condition.

**Table 2:** Production score means (standard deviations in parentheses) by condition and test session.

Condition	Session 1	Session 2	Total across sessions
Without book	0.25 (0.47)	0.50 (0.69)	0.76 (1.12)
With book	0.25 (0.58)	0.57 (1.09)	0.82 (1.41)

Given the generally low performance of participants across conditions and sessions in the retention task, to further probe whether there might be some differences between the two conditions that were masked by floor effects, we also conducted a nonparametric analysis of the identification scores for just those participants who scored above chance (> 2.0) on identification in the first test session (*n* = 17). This included 5 participants from the with book condition (36%) and 12 participants from the without book condition (60%). While proportionally more children performed above chance on monster name identification from the without book than from the with book condition, a Chi Square test revealed that these frequency differences were not statistically significant, 𝛸^2^ = 2.882, *p* = 0.0896 (two-tailed).

In addition, following the same procedure for the participants who scored above zero on the production task in the first session (*n* = 8), we found that only 3 children with books and 5 children without were able to name a monster themselves after hearing it labeled in the story, a nonsignificant frequency difference between the two conditions, 𝛸^2^ = 0.500, *p* = 0.4795 (two-tailed).

In sum, despite the descriptive appearance of a novel word retention advantage for children who were assigned to the without book condition, there were no significant differences in retention found between participants who did or did not have a paper copy of the story of their own with which to follow along.

### RQ 3: Does the addition of a physical book influence children’s engagement during SMSR?

**Figure 4:**
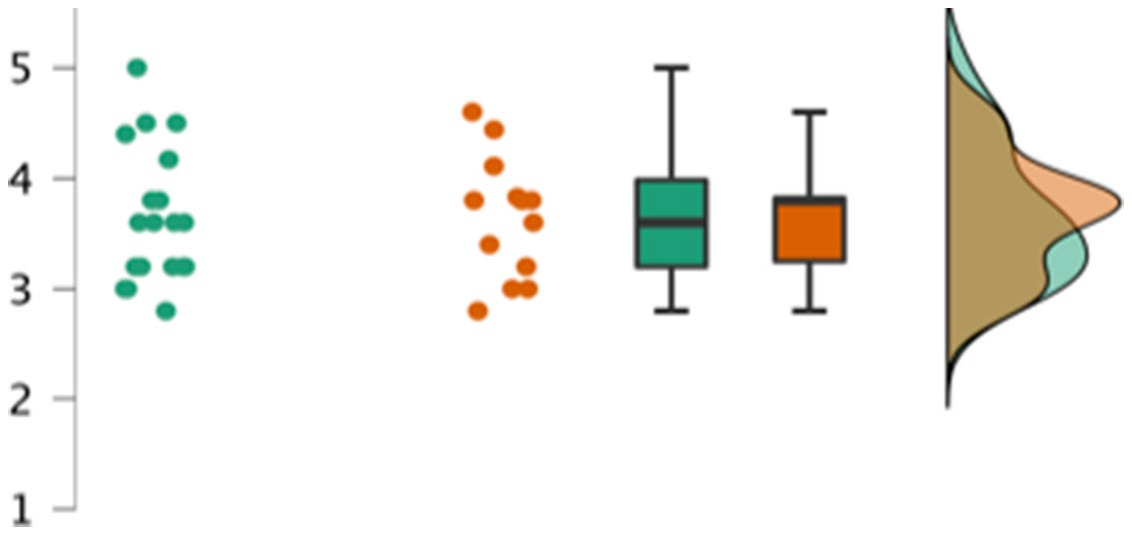
Participants’ average engagement scores by condition. In three alternative ways the distribution of engagement scores for participants are represented with dots, box plot, and curves. Green indicates the scores of participants in the ‘without book’ condition. Orange indicates scores for participants in the ‘with book’ condition.

In order to test whether there was a relationship between having a book to follow along with, and children’s overall engagement, an independent sample t-test was used to test differences between children’s average engagement scores by condition. No difference in average engagement scores were found between the with book (*M* = 3.66, *SD* = 0.53) and without book (*M* = 3.68, *SD* = 0.61) conditions, *t*(32) = 0.113, *p* = 0.911, *d* = 0.039. Thus, variability in children’s engagement in SMSR was not reliably related to whether or not they were provided with the additional paper copy of the story (Figure 4).

While our quantitative measure of participants’ average engagement while listening to SMSR did not show condition differences, when we looked closely at the content of children’s utterances and their non-verbal responses to the stories as they were heard, it was apparent that there were still qualitative differences between reading sessions where children did or did not have their own copy of the story. Those children who were provided with their own copy of the book often engaged in what we would describe as “matching up” behaviors and reactions. Often in this condition, when the researcher was reading about a monster via SMSR, children who had access to their own copy of the story would comment that they had found the monster in their own book (e.g., “oh yeah I have that one!,” “wait I cannot turn the page”), or nonverbally pointed and showed the researcher or a parent who was sitting nearby to them off-screen their own copy of the book with the matching monster. In fact, most verbalizations that were made by children in the ‘with book’ condition were about finding or trying to find the matching monster in their own copy of the story. Whereas, in the ‘without book’ condition children were also engaged in the SMSR, making verbal comments (e.g., “that one has antlers like this [makes antler ears with hands],” “a groze? I do not like groze.”) and nonverbally reacting directly with the monster presented on the screen and described by the researcher. Quantitatively, this summed to equal *amounts* of engagement in both conditions, while the *type* of engagement still appeared qualitatively different. Descriptively, children who had their own copy of the story with which to follow along, seemed both eager to engage with the activity, but also more distracted from the content of the story.

### RQ 4: Does children’s engagement during SMSR predict their ability to fast map new words from a story?

**Figure 5:**
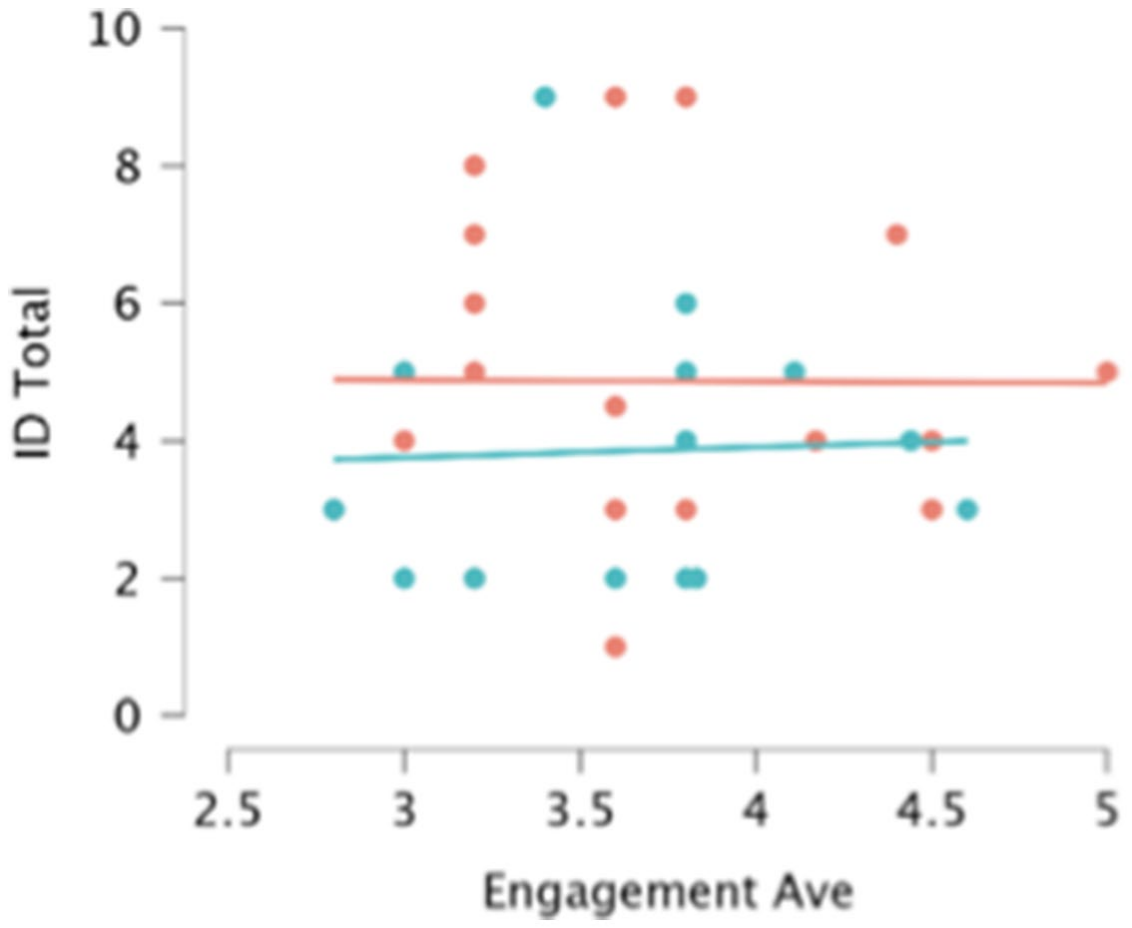
Participants’ total identification scores by average engagement scores. Green dots and regression line indicate the individual total identification (across both sessions) and average engagement scores of participants in the ‘without book’ condition. Orange dots and the regression line indicate scores for participants in the ‘with book’ condition.

Our final query into children’s levels of engagement in SMSR was whether engagement, even if unaffected by condition, was related overall to children’s likelihood of remembering the novel monsters that they heard in the story. However, there were no significant bivariate correlations between participants’ average engagement scores and either their correct identification or production scores in either session, *r*’s all <0.18, *p*’s > 0.320. Even when we combined children’s correct identification scores across both their first and second testing sessions, and partialed out condition, there was still no significant correlation between children’s engagement and identification, *r* = 0.011, *p* = 0.954 (see Figure 5).

In sum, variability across individual children in our general measure of engagement was not predicted by whether children had their own copy of the story, nor was it predictive of children’s success in remembering the novel monsters presented in the story.

## Discussion

### Summary of findings

The study described here was meant to be a simple replication of previous studies that have demonstrated that young children can easily fast map and remember new words from supportive shared reading experiences, set within an emerging medium – screen mediated shared reading (SMSR) over video-chat. This study revealed both how children’s engagement was affected by reading in an SMSR setting, as well as what new factors we must consider when measuring children’s retention of new information from SMSR. These data indicated that 3- to 5-year-old children’ novel word mapping and retention scores were slightly above chance. However, when compared to findings from previous in-person studies using the same story and same retention tasks (e.g., Read, 2014; Read et al., 2019), children’s new word retention in SMSR appeared to be more difficult. In addition, no correlations were found between children’s measured engagement levels and their mapping and retention of the new monster labels from the SMSR. Further, while children’s overall engagement and monster name retention did not quantitatively differ between conditions in which they either did or did not have their own paper copy of the book to follow along with, qualitatively children seemed less engaged with learning the monster names and more distracted by attempting to match-up their copy storybook with the reader’s shared screen in the condition where they had a printed version of their own.

These findings raise several questions to consider as we evaluate the benefits and challenges of screen mediated shared reading with young children.

### Why is fast mapping from SMSR challenging?

One of the main findings from this study in our view was that children appeared to perform less well on this novel monster name mapping and retention task via screen mediated reading than they typically have done in a very similar version of the task in in-person shared reading settings. This may not be because of something inherently challenging about the use of screens in shared reading, as several other studies have found successful learning outcomes from e-books and screen mediated reading (Lauricella et al., 2014; Gaudreau et al., 2020). Rather, the less than optimal performance in this study may be because of the additional attentional demands that SMSR may pose for young children compared to more traditional dyadic shared reading. For instance, there are new potential distractions for a child to manage when engaged in SMSR in a physical environment that is different from that of the reader. Additional sounds, movements, and other people in a child’s immediate environment can all draw attention away from the person and activity that they are engaged with on-screen. Due to the nature of video-chat, researchers have less control over the local environment of those participating. In this study, while many caregivers spontaneously made an effort to give their child a quiet space to participate, they also often set the child up in a space where they could be supervised and without headphones so that caregivers could see and hear if they needed technical support. These factors were helpful for supporting the child’s participation in the task, but may have also meant more potential for distraction. Support for the intuitive hypothesis that more in-home distraction can make learning over video-chat more challenging comes from other recent research on children’s learning during pandemic-related remote learning situations. When measuring the factors impacting the success of remote learning for school children, Chifari et al. (2021) found a latent variable, “impact on the family,” that affected engagement levels. During the first few weeks of lockdown, children’s engagement levels were lower if they needed to share a learning space, as well as technological resources, with their family. In addition, Chere and Kirkham (2021) found that older children and adolescents performed worse on executive functioning tasks when listening to audio simulating noisy home environments (e.g., “dog[s] barking, door[s] opening and closing, doorbell[s] ringing, traffic, birds, various toys”) than those who listened to simple, white noise audio. These simple demonstrations show how a child’s immediate environment can increase the attentional demands of learning tasks that happen over video-chat, even when video-chat is a more contingent and engaging medium than other screen-based learning (e.g., Glick et al., 2022).

Moreover, the potential distractions are not limited to the physical space around the child, but may also involve the use of and configuration of the screen mediated activity itself. While there was a baseline for participants in this study in their familiarity with video-chat in general, there was still potential for participants’ varied range of experiences with screen mediated shared reading. This varied familiarity could, in turn, have impacted their responses (and distraction around) SMSR. Just as in previous studies comparing e-book reading to traditional print book reading, we must consider the role that novelty of the task can play for young children (e.g., Parish-Morris et al., 2013; Rvachew et al., 2017). On the one hand, novelty of SMSR with a new researcher could increase general engagement and excitement about participating in the session, but on the other hand the novelty can also become a distraction that pulls the child away from focusing on the content of the reading. A child with less familiarity with shared reading over video-chat in addition to the novelty of the story and the reader may require more attentional resources, limiting their ability to focus on its contents. In addition, Lauricella et al. (2022) highlights a unique distraction to reading over Zoom – the ability to view one’s own face. Unless the child is overtly directed into ‘speaker mode’ at the beginning of a Zoom session, they likely have access to their own image throughout the session, which was the case in this study. According to Lauricella et al. (2022), children between ages 4 and 6 who had access to looking at themselves during Zoom spent more time looking at themselves (13% of the total activity time) than anyone or anything else in the Zoom, and thus performed worse on a story comprehension task. Overall, the more time children spent looking off screen, the worse their story comprehension.

Thus with many other places to look and other possible distractions present in the immediate environment competing for preschool-aged children’s attentional resources, success in learning from SMSR of this kind may be more sensitive to many developmental factors. Children in this age range have varied and developing levels of attentional control, working memory, and language processing and comprehension skills; any or all of which could impact their success in this kind of fast-mapping task in a noisy environment. While an important limitation of the current study was that we were not able to collect measures of participants’ individual differences in cognitive development, we did find that children’s age in months, which is typically correlated with development in language, memory, and attentional skills, was positively correlated with their overall retention of new words from the stories. Thus, future research including additional measures could help parse out the effects of these factors on children’s potential for learning in situations like SMSR where the potential for environmental distraction is high.

Children may also have been less successful at the specific task of fast-mapping new words in a video-chat setting compared to prior in-person reading studies, because of the focused attention to unique word-picture pairings that was required within a more potentially distracting environment. Even though other recent screen based shared reading research has found more promising results, our 8-item 4-alternative-choice vocabulary test may have been more challenging than other measures of vocabulary retention and story comprehension used in recent studies (e.g., Gaudreau et al., 2020). Given this limitation of the current study, in future research it would be useful to probe different degrees of memory for both the vocabulary and the content of a story for a clearer understanding of what children can learn in this modality, rather than relying on a single difficult task.

### Why did not engagement correlate with learning?

In the current study, to our surprise no correlations were found between children’s measured engagement levels and their mapping and retention of the new monster names from the SMSR. This raises the question of whether engagement is truly disassociated from learning in this setting, or perhaps more likely, our measure of engagement may not be sensitive enough to capture such a link.

Engagement should generally be a positive correlate of learning. Certainly attending to and interacting with new ideas, like the names of novel monsters, should help them be maintained in a child’s memory. However, it is also likely that the *type* of engagement matters for the specific goal of any learning task. In an e-book reading study by Lauricella et al. (2014) engagement was measured as a composite of parent *and* child mutually reinforcing behaviors, and positively predicted learning from the stories that were shared. In the current study, engagement was measured differently, and how children engaged in SMSR differed individually. Some were verbally engaging with the reader, apparently trying to make a connection by pointing out features of or asking questions about the monsters described in the story she was reading. Some children were physically engaged with the book (if they had their own copy), flipping ahead of the page and playing with the paper. Other children appeared to be only passively engaging, listening quietly and observing the reader through the screen.

In addition, it is of note that in our study, participants were hearing the story read by an unfamiliar researcher, who was intentionally *not* engaging in extra-textual dialogic reading practices (i.e., prompting the child to comment on the story with questions, or expanding on comments the child might make) either during or after the story read aloud. This was, of course, meant to control the reading experience to make it as similar as possible for each child regardless of condition, but it also became a limitation both to the ecological validity of the SMSR as well as to the support for engagement and learning available to the children. Measuring overall engagement may not capture the nuances or consequences of these different approaches to SMSR. For instance, listening carefully or repeating monster names may help children remember them, but engaging physically with the book, matching behaviors, or laughing and smiling a lot might all be signs of engagement with the activity but distraction from the subtask of vocabulary learning.

Engagement is a challenging construct to operationalize especially in this context of SMSR. Although an adapted version of the Leuven Scale of Active Engagement (e.g., Lingwood et al., 2021) was used as the primary measure of engagement in our study, it is typically a resource for in-person activities. Measuring engagement through the confines of a Zoom video box was limited; our narrowed view of the scene meant that anything occurring behind the camera that could potentially interfere with the child’s level of attention was missed. In many cases, this called into question whether or not the child was actually looking at the screen and following along attentively with the story. It is possible that while the story was being read to them, they were preoccupied with something slightly above the camera in their line of sight but not visible to the researcher. In addition, using verbal utterances as evidence of engagement also comes with limitations. Children have varying levels of comfort, especially in this somewhat novel task of screen mediated reading with an unfamiliar person. Some of our participants may have been more shy than others causing them to sit through the reading session without saying anything in response to the story. However, this does not necessarily mean that they were not as engaged as another participant with a high amount of verbal utterances. As a new mode of reading, SMSR may pose new challenges to measuring engagement due to the uncertainties surrounding how each child will respond in their respective environments within the conditions they were placed in. In this study, these limitations likely affected our ability to truly determine whether or not a child was “fully engaged” and thus our measure may not have been sensitive enough to capture a link between engagement and new word retention.

### What factors could change children’s success in learning from SMSR?

In the current study, we compared two different types of screen mediated shared reading – screen sharing an e-book while children either had their own paper copy to follow along with or not. There are, however, other configurations for SMSR that may impact both children’s overall engagement and attention to specific aspects of the shared reading that could promote specific types of learning. For example, according to preliminary findings in a study by Strouse et al. (2023) of 3- to 5-year-olds engaged in SMSR, differences in set-up (i.e., holding a copy of the physical storybook up to the camera while the child did or did not have their own copy vs. screen sharing an e-version) could have differing impacts on children’s joint attention to the environment of the researcher and engagement behaviors. Strouse et al. (2023) found that the best configuration for *engaging with* SMSR seems to be the two-book condition where the reader and child both have physical copies of the book, as in this case there is more across-screen attention. However, in their study, it is not clear if this configuration would also promote the highest level of learning, especially in the form of a demanding novel word learning task. Further research into the impacts of various configurations with and without physical copies of the books or screen-sharing, is needed to untangle the impacts of how children engage with SMSR and what they ultimately can attend to and learn from it.

### Is SMSR still a good option?

Whether or not SMSR is truly beneficial depends on several factors, one being the purpose of SMSR in the first place. SMSR is used when reading with a child is desired, but when physical proximity is not possible, i.e., it seems to largely be the “best alternative” when in-person shared reading is not an option. Thus, when considering its effectiveness, we must carefully consider what we are comparing it to and what is meant to be its purpose.

Shared reading in general is a common activity in children’s daily lives that serves many purposes, not always pedagogical. While children can and do learn new words from shared reading, often the purpose behind reading a book with a child goes beyond vocabulary building. Shared reading can promote adult-child interaction and narrative development (Gilkerson et al., 2017), preliteracy skills (Reese and Cox, 1999), and even simply provide a shared experience between a caregiver and a child that can be linked to reduced stress and healthy attachment (Bus and van Ijzendoorn, 1997; Canfield et al., 2020). Thus, even if SMSR may be a more challenging way of teaching vocabulary from stories compared to in-person reading, that does not mean it should be ruled out in terms of providing benefits in these other domains. Furthermore, when compared to other forms of e-reading, such as watching pre-recorded story presentations, or engaging with an e-book, live synchronous contingent interaction with a caregiver is still more engaging and often preferred by both the child and the readers (e.g., Strouse et al., 2021; Glick et al., 2022), and so while SMSR may need to be configured and optimized further to best suit the goal of vocabulary teaching through shared reading, it is clearly a positive addition to the options available to children with books and all that can be learned from them.

## Data availability statement

The raw data supporting the conclusions of this article will be made available by the authors, without undue reservation.

## Ethics statement

The studies involving human participants were reviewed and approved by Santa Clara University Institutional Review Board for Social Behavioral Educational Research. Written informed consent to participate in this study was provided by the participants’ legal guardian/next of kin. Written informed consent was obtained from the individual(s), and minor(s)’ legal guardian/next of kin, for the publication of any potentially identifiable images or data included in this article.

## Author contributions

KR was the lead faculty supervisor on the project. KR and HH lead the writing of this manuscript with expert contributions and editing from BM and KY. All authors contributed to planning, running and analyzing the findings of the study.

## Conflict of interest

The authors declare that the research was conducted in the absence of any commercial or financial relationships that could be construed as a potential conflict of interest.

## Publisher’s note

All claims expressed in this article are solely those of the authors and do not necessarily represent those of their affiliated organizations, or those of the publisher, the editors and the reviewers. Any product that may be evaluated in this article, or claim that may be made by its manufacturer, is not guaranteed or endorsed by the publisher.
